# Insular Organization of Gene Space in Grass Genomes

**DOI:** 10.1371/journal.pone.0054101

**Published:** 2013-01-11

**Authors:** Andrea Gottlieb, Hans-Georg Müller, Alicia N. Massa, Humphrey Wanjugi, Karin R. Deal, Frank M. You, Xiangyang Xu, Yong Q. Gu, Ming-Cheng Luo, Olin D. Anderson, Agnes P. Chan, Pablo Rabinowicz, Katrien M. Devos, Jan Dvorak

**Affiliations:** 1 Department of Statistics, University of California Davis, Davis, California, United States of America; 2 Institute of Plant Breeding, Genetics and Genomics (Department of Crop and Soil Sciences), Department of Plant Biology, University of Georgia, Athens, Georgia, United States of America; 3 USDA/ARS Western Research Center, Albany, California, United States of America; 4 Department of Plant Sciences, University of California Davis, Davis, California, United States of America; 5 The J. Craig Venter Institute, Rockville, Maryland, United States of America; 6 Institute for Genome Sciences, and Department of Biochemistry and Molecular Biology, University of Maryland School of Medicine, Baltimore, Maryland, United States of America; National Institute of Environmental and Health Sciences, United States of America

## Abstract

Wheat and maize genes were hypothesized to be clustered into islands but the hypothesis was not statistically tested. The hypothesis is statistically tested here in four grass species differing in genome size, *Brachypodium distachyon*, *Oryza sativa*, *Sorghum bicolor*, and *Aegilops tauschii*. Density functions obtained under a model where gene locations follow a homogeneous Poisson process and thus are not clustered are compared with a model-free situation quantified through a non-parametric density estimate. A simple homogeneous Poisson model for gene locations is not rejected for the small *O. sativa* and *B. distachyon* genomes, indicating that genes are distributed largely uniformly in those species, but is rejected for the larger *S. bicolor* and *Ae. tauschii* genomes, providing evidence for clustering of genes into islands. It is proposed to call the gene islands “gene insulae” to distinguish them from other types of gene clustering that have been proposed. An average *S. bicolor* and *Ae. tauschii* insula is estimated to contain 3.7 and 3.9 genes with an average intergenic distance within an insula of 2.1 and 16.5 kb, respectively. Inter-insular distances are greater than 8 and 81 kb and average 15.1 and 205 kb, in *S. bicolor* and *Ae. tauschii,* respectively. A greater gene density observed in the distal regions of the *Ae. tauschii* chromosomes is shown to be primarily caused by shortening of inter-insular distances. The comparison of the four grass genomes suggests that gene locations are largely a function of a homogeneous Poisson process in small genomes. Nonrandom insertions of LTR retroelements during genome expansion creates gene insulae, which become less dense and further apart with the increase in genome size. High concordance in relative lengths of orthologous intergenic distances among the investigated genomes including the maize genome suggests functional constraints on gene distribution in the grass genomes.

## Introduction

Genes in grass genomes are separated from each other by intergenic regions often containing transposable elements (TEs) [1]. The balance between the rate with which TEs are inserted into an intergenic region and the rate with which they are deleted determines whether the region is expanding or contracting [1]–[4]. Changes in this balance are almost certainly the primary cause of variation in genome size and gene density along chromosomes.

Gene density in many grass genomes increases overall from the proximal towards the distal regions of chromosome arms. This gradient is heterogeneous and regions of higher and lower gene density are superimposed on it. This pattern of gene distribution has been observed in all grass genomes sequenced to date [5]–[10] and was also inferred in wheat chromosomes by deletion mapping [11]. Heterogeneity in gene distribution along wheat chromosomes has been used to hypothesize that wheat genes are clustered into a limited number of very large gene islands separated by vast expanses of gene-empty space [12], [13]. Gene distribution on BAC-based physical maps of wheat chromosome 3B and the seven chromosomes of *Aegilops tauschii,* the diploid source of the wheat D genome [14], [15], failed to support this hypothesis [16], [17]. Sequencing of single wheat BAC clones and BAC clone contigs suggested that gene clustering into many small islands is a more likely scenario [18]–[20]. Sequencing and annotation of BAC contigs from wheat chromosome 3B totaling 18.2 Mb revealed a chromosome-wide average gene density of 1 gene per 96 kb but a frequency distribution of intergenic distances showed a peak of very short intergenic distances [20]. This peak was used as evidence for gene clustering into small gene islands [20]. A N50 (median) distance between genes of 43 kb was arbitrarily chosen as the upper limit for intergenic distances within gene islands. Using this criterion, 75% of genes and pseudogenes were contained within 42 gene islands in the 18.2 Mb region of chromosome 3B. The islands contained on average 3.2 genes.

A similar pattern has also been reported in the maize genome. Analysis of two proximal regions in maize chromosome 3 revealed gene islands containing from 1 to 4 genes separated by clusters of long-terminal repeat (LTR) retroelements [21]. These LTR retroelement clusters ranged from 23 to 155 kb [21]. While the overall gene density was estimated to be 1 gene per 111 kb, a gene density of 1 gene per 16 kb was observed within gene islands. Analyses of a larger portion of the maize genome showed that about half of the intergenic distances in maize were shorter than 20 kb [22].

All studies of gene clustering in grass genomes have been intuitive and lacked statistical inference. It cannot be excluded in the absence of a formal statistical analysis that the distribution of distances between genes, such as that described by Choulet et al. [20], is typical for genes that are distributed uniformly along a chromosome.

In order to gain better understanding of the structure and evolution of gene space in grass genomes, we obtained statistical inference for the null hypothesis that gene locations in orthologous regions of the genomes of four grass (Poaceae) species, *Sorghum bicolor* (sorghum), *Oryza sativa* (rice), *Brachypodium distachyon* (false brome), and *Ae. tauschii* (goat grass), follow a homogeneous Poisson process. *Aegilops tauschii* belongs to the tribe Triticeae, and like all members of the tribe it has a large genome (4,020 Mb [23]). The sorghum genome at 730 Mb [7] is about a one-sixth of the *Ae. tauschii* genome. The remaining two species, rice and *B. distachyon,* have genomes more than an order of magnitude smaller than the *Ae. tauschii* genome; the rice genome is 389 Mb [5] and that of *B. distachyon* is 271 Mb [9]. By comparing four genomes differing in size, we assess the effects of genome expansion on the evolution of gene space and gene distribution in grass genomes.

## Materials and Methods

### Genome Sequences

Since the *Ae. tauschii* genome has not been sequenced, sequences of nine randomly selected megabase-size contigs of BAC clones totaling 9,796 kb were generated for this study [24]. The contigs were located in four of the seven *Ae. tauschii* chromosomes and originated from both distal and proximal chromosome regions. A total of 90 genes were manually annotated in the *Ae. tauschii* contigs suggesting an average gene density (excluding pseudogenes) of 1 gene/106 kb and predicting a total of 36,371 genes in the genome [24]. These estimates are close to those inferred for the wheat B genome on the basis of chromosome 3B BAC contig sequencing [20].

The orthologous regions in *B. distachyon,* rice and sorghum had been shown to harbor 62, 67 and 72 genes, respectively [24]. In all species analyzed, intergenic distances were measured as the distance between the start/end of the coding region of neighboring genes.

Because the *Ae. tauschii* BAC sequence consisted of, on average, 4.5 scaffolds per BAC [24] and the size of the gaps between scaffolds was unknown, we considered scaffolds as contiguous (that is, no extra bases were added to account for the sequence gaps) for the purpose of calculating the intergenic distances. This is unlikely to affect our analysis as the majority of small intergenic distances analyzed here (97% of intergenic distances ≤20 kb) were derived from genes located on the same scaffold. Also, although 64% of sequence scaffolds within a BAC clone could be ordered based on the overlap with neighboring BAC clones [24], some genes were located on unordered scaffolds. For those genes, we determined both the smallest and largest distance to the nearest gene considering all possible contig orders, and used the average between the two distance measurements in our statistical analyses (Table S1). For genes that were located on different BAC clones, intergenic distances were calculated as described above, but taking into account the overlap between neighboring BAC clones. To analyze the distribution of intergenic distances, all genes annotated in the orthologous regions in *Ae. tauschii*, *B. distachyon*, rice, and sorghum were taken into account (Tables S2, S3, S4). To analyze the level of gene island conservation across species, we only considered orthologous gene pairs that were present in all four species analyzed (Table S5).

### Distribution of Intergenic Distances

An initial step in the analysis of gene density in the grass genomes is to formally test the pertinent null hypothesis that genes are randomly located along the chromosomes according to a uniform distribution. This distribution will arise if the locations of the genes along the chromosome follow a homogeneous Poisson process [25]. Under this model, gene locations are independent of one another and the expected number of genes in any one region is simply proportional to the length of that region.

A consequence of the gene locations following a homogeneous Poisson process is that the density of intergenic distances will follow an exponential distribution. This implies that rather than directly testing for the Poisson process or the uniform distribution conditional on the total number of genes, we can test for the exponential distribution using the aggregated sample of intergenic distances from the 9 sequenced contigs, viewing the collection as a random sample from the population of all intergenic distances in the genome.

For each of the four species, denoting the density function of the distribution of intergenic distances by 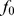, we tested the null hypothesis.*H_0_*: The intergenic distances follow an exponential distribution, *i.e.*

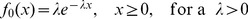
(1)
*vs H_A_*: The intergenic distances do not follow an exponential distribution.



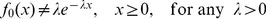
(2)This test can be implemented with a chi-square goodness of fit test using 10 cells with varying bin widths. This leads to expected frequencies ranging from 5 to 10 per cell, depending on the species. Under *H_0_*, the rate parameter *λ* was estimated using the maximum likelihood method. The applicable null distribution is a chi-square distribution with 8 degrees of freedom.

### Intergenic Distances Density Estimation

An estimate of the true density of intergenic distances was achieved via non-parametric density estimation. We employed a binning approach, first binning the raw data then applying a local linear kernel smoother on the resulting histograms [26]. The initial histogram density estimate is defined in the following way: Given the random sample 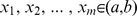 from the density of interest 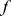, define an equally spaced partition of 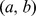 by 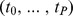 where 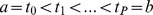. The bin width 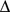 is determined by the number of bins so that 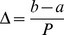. We use 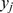 to denote the count of sample points falling within bin 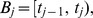 and 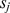 to denote the midpoint of 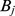 for 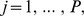 so that 
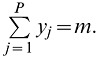
 The histogram density estimate 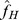 of 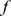 is given by
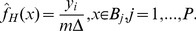
(3)


We obtain 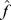 from 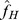 via local linear smoothing methods [27]. Formally, the estimate 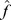 is obtained by minimizing the local weighted sums of squares,
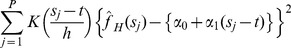
(4)for each 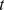 with respect to 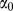 and 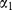 from which we obtain 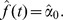 We used a Gaussian kernel 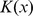 and chose the bandwidth 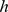 manually.

### Conservation of Gene Islands Across Species

Gene pairs were used to evaluate the preservation of gene islands across species. For the purposes of this analysis we defined a gene pair as two genes that were neighbors in the ancestral grass genome [24] and that were both present in the analyzed species *Ae. tauschii, B. distachyon*, rice and sorghum. For each pair, we obtained the distance between the neighbors in each of the four species. In addition to 28 gene pairs identified from the *Ae. tauschii* data set, we also identified 8 gene pairs from sequenced BAC clones of hexaploid wheat cultivar Chinese Spring (J.L. Bennetzen and K.M. Devos, unpublished data) with orthologs in *B. distachyon,* rice and sorghum. In order to evaluate the preservation of gene islands across species we calculated Kendall's coefficient of concordance W [28], [29] using a correction for ties since the original data consists of raw distances rather than ranks. This statistic was then used to test significance of the coefficient of concordance by testing the null hypothesis 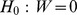.

### Correlation of Distances between Neighboring Genes and Location of Contig on the Centromere-telomere Axis

The relative location of each contig on the centromere-telomere axis, ranging from 0 (centromere) to 1.0 (telomere), was downloaded from the *Ae. tauschii* genetic maps reported by Massa et al. [24]. Distances from the end of a gene to the beginning of the neighboring gene in nucleotides were determined for all nine contigs. Only distances between genes were measured; distances from the first and last gene to the respective contig edge were disregarded. For each contig, the empirical quantile function of intergenic distances was computed and distances within the first quartile were considered intra-insular distances and distances within the fourth quartile were considered inter-insular distances. In order to determine if a relationship exists between location on the centromere-telomere axis and gene island structure, we implemented the regression model:
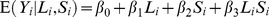
(5)where 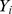 is the intergenic distance between the *i*th pair of genes, 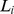 is the location along the centromere-telomere axis of the *i*th gene pair and 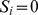 if the pair is categorized as intra-insular and 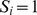 if the pair is categorized as inter-insular by the above quantile approach. For intra-insular distances, 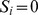, and the model is
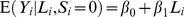
(6)where 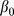 represents the intercept and 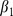 is the slope describing the linear relationship between intra-insular distances and location. For inter-insular distances, 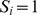, and the model is
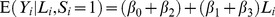
(7)where 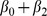 represents the intercept and 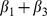 is the slope describing the linear relationship between inter-insular distances and location. This model allows for formal testing of the following two null hypotheses:

#### Hypothesis 1


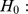
 Intra-insular distances within islands are homogeneous along the *Ae. tauschii* chromosomes irrespective of location along the centromere-telomere axis 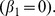


#### Hypothesis 2


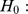
 Inter-insular distances are homogeneous along the *Ae. tauschii* chromosomes irrespective of location along the centromere-telomere axis 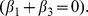


### Repeat Elements in Intergenic Regions

All intergenic regions in the nine *Ae. tauschii* contigs that were 100 kb or less in length were analyzed for the presence of LTR retrotransposons by BLASTN searches against the Triticeae Repeat (TREP) database (http://wheat.pw.usda.gov/ITMI/Repeats/). We also conducted a BLASTN search of the intergenic regions against Triticeae sequences in the ‘High Throughput Genomic Sequences’ (HTGS) section of GenBank, which contains several hundred sequenced wheat and *Ae. tauschii* BAC clones. Intergenic regions with multiple hits were annotated according to the annotation found in the BAC clones deposited in GenBank. Hits with a minimum length of 50 bp and an e-value ≤1e^−10^ were considered significant (Table S1). LTR retrotransposons in rice intergenic regions were identified using the same criteria by BLASTN searches against the repeat database ISU Cereal Repeat DB 3.1 (http://magi.plantgenomics.iastate.edu) (Table S2).

## Results

### Density Functions of Intergenic Distances

**Figure 1:**
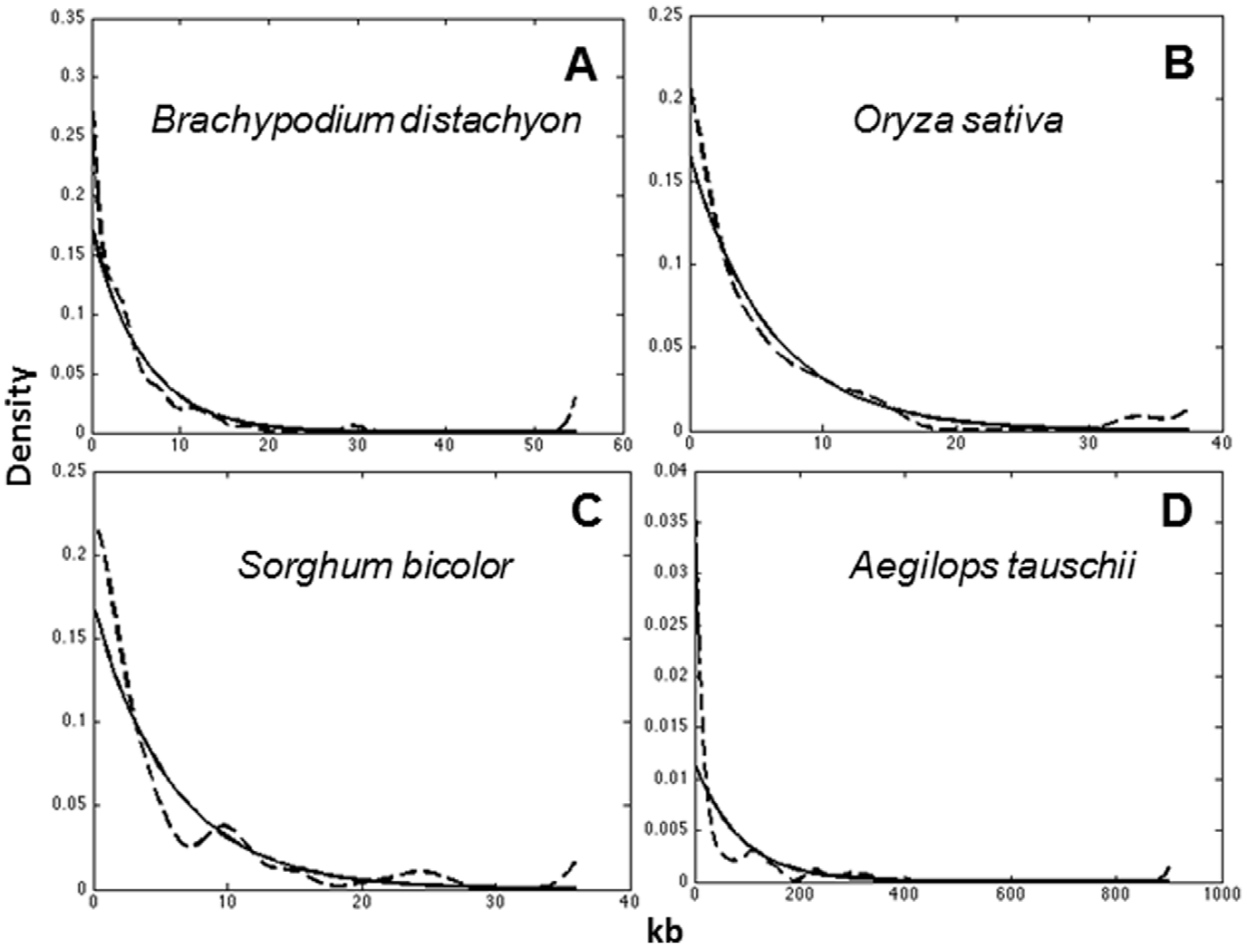
Density functions for intergenic distances in *B. distachyon* (A), rice (B), sorghum (C), and *Ae. tauschii* (D). Shown is the exponential density fitted by Maximum Likelihood (2) (solid) and the non-parametric density estimate (4) (dashed), with bandwidth *h* = 1.25 (**A**), *h* = 1.75 (**B**), *h* = 1.50 (**C**), and *h* = 15.00 (**D**).

The graphs of the non-parametrically estimated density functions (dashed lines in Figure 1) show that short distances between neighboring genes are the most frequent distances in all four genomes. Short distances are also most frequent in the graphs of gene locations as captured by the null hypothesis of uniform locations of genes along chromosome arms given by Equation (1) (solid lines in Figure 1). In *B. distachyon* and rice, the non-parametric density functions do not significantly differ from the fitted exponential density for those species (Figures 1A and 1B and Table 1) and indicate that genes are not clustered in these species. In contrast, in sorghum and *Ae. tauschii,* non-parametric density functions significantly differ from the fitted exponential density and have several features in common (Figures 1C and 1D and Table 1). In both genomes, the non-parametric density graph is above the fitted exponential density graph for short intergenic distances, indicating the presence of too many short distances, and then below it, indicating too few intermediate distances. This pattern suggests that gene clustering is present. We propose to call these clusters “gene insulae” and the pattern of gene distribution observed in the sorghum and *Ae. tauschii* genomes as “insular gene distribution”. We propose this new term because the English term “gene islands” has been used for other forms of gene clustering (see Introduction).

**Table 1:** Summary of test results for the null hypothesis that gene locations are uniformly distributed in the four species.

	No. intergenic distances	Estimated rate parameter 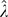 *	Estimated standard error of 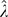	*P*-value**
*B. distachyon*	52	0.174	0.024	0.330
Rice	57	0.167	0.022	0.064
Sorghum	62	0.170	0.022	0.007
*Ae. tauschii*	81	0.012	0.001	0.000

* The estimated exponential rate parameter 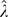 is the maximum likelihood estimator of 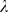 as given in Equation (1).

** Based on a χ^2^- goodness of fit test.

The sorghum and *Ae. tauschii* non-parametric density graphs display an initial interval of very short intergenic distances on which the estimated non-parametric density function is concave down (Figures 1C and 1D). This portion of the graphs intersects the density function based on the null hypothesis at 3 kb and 22 kb in sorghum and *Ae. tauschii,* respectively, passes through a local minimum at 8 and 81 kb, respectively, changes to concave up, again intersects the function based on the null hypothesis and reaches a local maximum, with one or more additional concavity changes as the intergenic distances increase (Table 2). We propose using the first local minimum to separate the intra-insular intergenic distances on the left from inter-insular distances on the right. This choice is motivated by cluster analysis based on non-parametric density estimation [30]. The mean intergenic distance shorter than this minimum (*i.e.* the mean intra-insular distance) is 2.1 kb in sorghum and the average insula contains 3.7 genes. The mean intergenic distance shorter than this minimum is 16.7 kb in *Ae. tauschii* and the average insula contains 3.9 genes. The average intergenic distance longer than the first local minimum (*i.e.* the mean inter-insular distance) is 15.1 kb in the sorghum genome but is significantly larger at 205.2 kb in the *Ae. tauschii* genome (*Mann-Whitney Test*, *P*-value = 0). The distances shorter than this critical point represent 70% and 63% of all intergenic distances in the sorghum and *Ae. tauschii* genomes, respectively.

**Table 2:** Insular structure in sorghum and *Ae. tauschii.*

Statistic	Unit	Sorghum	*Ae. tauschii*
First local minimum	kb	8.0	81.0
Mean of distances shorter than the minimum	kb	2.1	16.7
Mean of distances longer than theminimum	kb	15.1	205.2
Shorter distances	%	70.0	63.0

### Gene Density along the Centromere-telomere Axis

**Figure 2:**
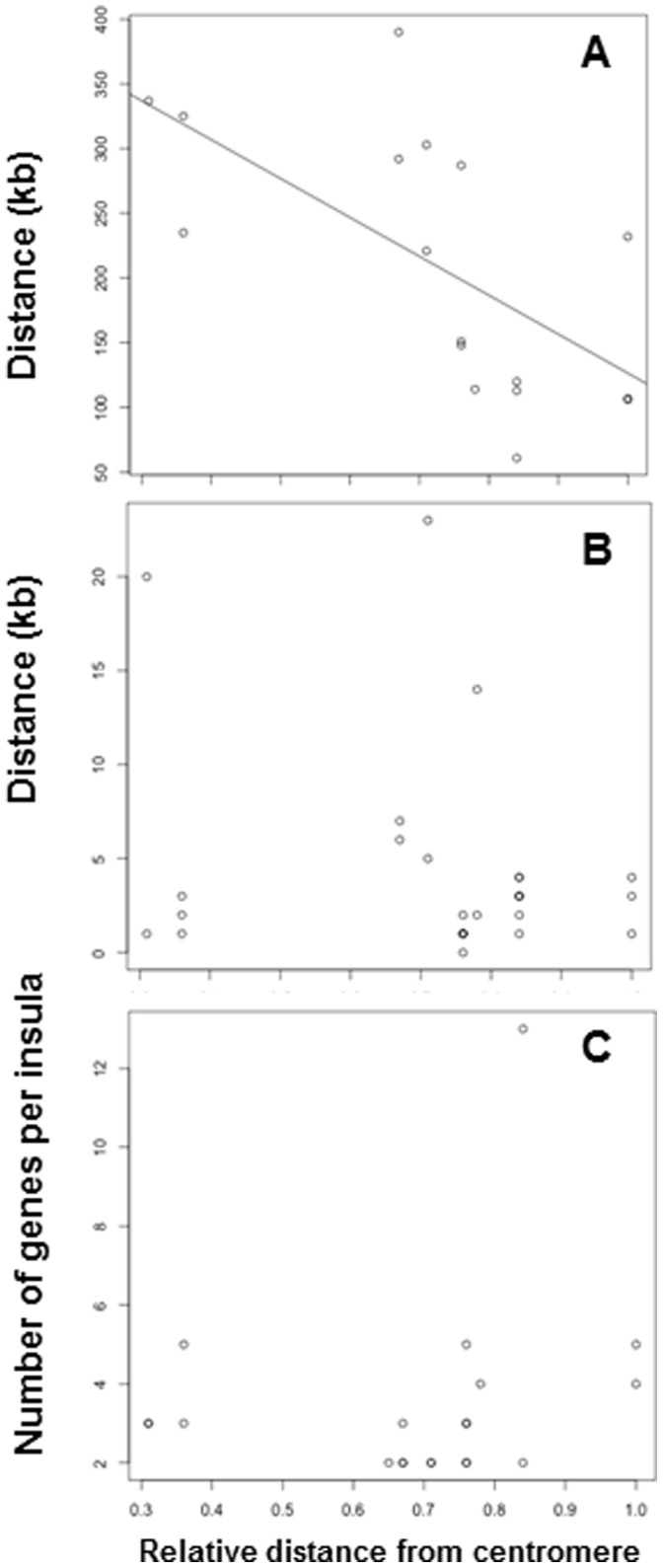
Relationships between inter-insular distances (A), intra-insular distances (B) and the number of genes per insula (C) and gene location along the centromere-telomere axes of *Ae. tauschii* chromosome arms. (**A**) shows a fitted regression line from Model (7) of the inter-insular distances and gene location on centromere-telomere axes of *Ae. tauschii* chromosome arms.

Gene density increases from the proximal, low-recombination regions towards the distal, high-recombination regions in the *Ae. tauschii* chromosomes [17]. This gradient could be caused by a change in gene density within gene insulae, a change in the distances between gene insulae, or both. In order to determine which of these three possibilities is most consistent with the data, we fit the regression model in (5) to *Ae. tauschii* data after removing a single outlier due to its high influence on the parameter estimates. The fitted regression model (5) indicates no relationship between intra-insular distances and gene location along the centromere-telomere axis based on the high *P* values for parameters 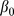 and 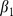 (Table 3). Since 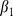 cannot be distinguished from zero at the chosen significance level, the significance of 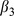 indicates that while there is no significant relationship between intra-insular distances and insula location along the centromere-telomere axis there is a significant relationship between inter-insular distances and location along the centromere-telomere axis (Figures 2A and 2B). This relationship between inter-insular distances and gene location is quantified in equation (7) and results are shown in Table 4. The fitted regression model (7) indicates that there is a strong negative relationship between inter-insular distances and insula location along the centromere-telomere axis (Figure 2A). We therefore conclude that increase in gene density towards the ends of *Ae. tauschii* chromosomes is caused primarily by a decrease in the distances between insulae.

**Table 3:** Fitting the full regression model specified in equation (5) to the *Ae. tauschii* data.

Parameter	Estimate	*P*-value	95% confidence interval
*_β_*_0_	8.3	0.82	(−64.5, 81.1)
*_β_*_1_	−5.5	0.92	(−102.8, 91.9)
*β*_2_	387.8	∼0.00	(279.9, 495.7)
*_β_*_3_	−257.8	∼0.00	(−403.0, −112.5)

**Table 4:** Fitting the regression model (7) to the *Ae. tauschii* data.

Parameter	Estimate	*P*-value	95% confidence interval
*_β_*_0+*β*2_	427.2	∼0.00	(274.3, 580.1)
*_β_*_1+*β*3_	−300.8	0.01	(−502.9, −98.6)

It should be noted that as an alternative to the quantile approach to classify intergenic distances as intra-insular or inter-insular used here, we could have used the first local minimum at 81 kb as the boundary between intra-insular and inter-insular distances. In fact, we implemented this method as well and it yielded identical inferences. We report the results of the quantile approach because this method is particularly robust. Similarly, we tried several variance stabilizing transformations prior to fitting the regression model. In all cases, the inferences were identical. We therefore report here the most parsimonious model. Clearly there is a strong signal in the data indicating a negative relationship between inter-insular distances and insula location along the centromere-telomere axis.

To determine if there is a relationship between the insula size and its location on the centromere-telomere axis in *Ae. tauschii*, we plot the number of genes per insula and insula location on the centromere-telomere axis for 15 *Ae. tauschii* insulae (Figure 2C). There is no relationship between the number of genes per insula and insula location (*r = *0.19, *P*-value = 0.42). The situation does not change after an obvious outlier (insula with 13 genes) is removed, (*r = *0.06, *P*-value = 0.8). We conclude that the sizes of insulae do not appreciably change along the centromere-telomere axis in the *Ae. tauschii* genome.

### Conservation of gene Insulae

Kendall’s coefficient of concordance W = 0.49, which indicates that there is a high level of concordance of distances between orthologous gene pairs in the four genomes. This means that gene pairs that are relatively close to one another in one genome are likely to be also close to one another in other genomes. Analogously, gene pairs that are relatively distant from each other in one genome are likely to be also far apart in the other three genomes. The null hypothesis that the intergenic distances are independent across genomes is rejected with *P*<0.001.

### Gene Insulae and LTR Retroelements

In the *Ae. tauschii* genome, all inter-insular intergenic distances (distances >81 kb) contain LTR retrotransposons but only 27% of intra-insular intergenic distances contain LTR retrotransposons or their remnants. As shown in Figures 1C and 1D, most of the intra-insular distances are overrepresented relative to the uniform gene distribution. In the *Ae. tauschii* genome, only 9% these intergenic distance (distances ≤22 kb, Figure 1D) contain LTR retrotransposon remnants (Table S6), which indicates that the absence of LTR retrotransposon insertions is the primary contributor to the overrepresentation of short intergenic distances and an important attribute of insulae in the *Ae. tauschii* genome.

## Discussion

An important feature of the density functions based on the null hypothesis is a prominent peak of very short intergenic distances predicted for each of the four genomes. Thus, very short intergenic distances are expected to dominate density profiles of observed intergenic distances even if gene locations were distributed uniformly along chromosomes. This fact is exemplified by the *B. distachyon* and rice genomes in which short intergenic distances dominate the density profiles, yet genes are not clustered in those genomes. Observation of a prominent peak of short distances in an empirical density profile, such as that observed by Choulet et al. [20] is not in itself sufficient to draw a conclusion regarding the existence of gene clustering.

Comparisons of the density functions based on the null hypothesis with the non-parametric density functions estimated from observed distances between neighboring genes divided the four genomes into two contrasting pairs. The first pair consisted of the small *B. distachyon* and rice genomes. In those genomes the nonparametric and exponential density functions did not significantly differ from each other. The second pair consisted of the larger sorghum and *Ae. tauschii* genomes, for which the two density functions differed significantly.

Variation in genome size in grasses is principally caused by the accumulation or loss of TEs. Genome expansion takes place principally by the accumulation of LTR retroelements, which have the tendency to insert into LTR retroelements resident in the chromosomes [1], [2], [4]. Genome contraction takes place principally by DNA deletion caused by unequal homologous recombination between LTRs of the same or related LTR retrotransposons and by illegitimate recombination within TE and other non-essential DNA [3]. Most of the observations that emerged here can be attributed to TE dynamics and the nonrandom insertion of LTR retroelements. In small, gene-dense genomes, such as those of *B. distachyon* and rice, the overall retroelement content is lower, 21.4% in *B. distachyon* [9] and 26% in rice [5], than in the larger genomes, 54% in sorghum and at least 51% in the *Ae. tauschii* genome [31]. Genes are distributed uniformly and there is very little gene clustering in the small genomes. In the nine studied regions, LTR retrotransposons or remnants of LTR retrotransposons were present in only 12% of rice gene pairs (Table S2) compared to 54% of *Ae. tauschii* gene pairs (Table S1). When comparing only orthologous gene pairs, 12% of the rice gene pairs but 61% of the *Ae. tauschii* gene pairs were separated by sequences with homology to LTR retrotransposons (Table S6). All of the *Ae. tauschii* inter-insular spaces contained retrotransposons. This is consistent with the hypothesis that expansion of grass genomes, which takes place predominantly by the accumulation of LTR retroelements [2], occurs principally in regions already containing LTR retroelements. As a result of this nested insertion of retroelements, genes that are separated by LTR retroelements will be pushed further apart from each other during genome expansion, which will create large arrays of LTR retroelements characteristic of inter-insular space [20], [21]. Genes that are not separated by LTR retroelements will tend to remain near each other and form insulae. Due to the LTR retroelement dynamics, gene distribution is largely homogeneous in small grass genomes but assumes insular organization as a consequence of genome expansion. As illustrated by the comparison of orthologous regions in the sorghum and *Ae. tauschii* genomes, insulae become less gene dense and separated by greater spans of nested TEs as a genome expands.

The distal chromosome regions are typically more gene-dense than the proximal chromosome regions in the Triticeae chromosomes. Regression analysis showed that gene number per insula and gene density within an insula were similar along *Ae. tauschii* chromosomes but that inter-insular distances were shorter in distal, high-recombination regions compared to proximal, low-recombination regions. Therefore, the increase in gene density toward the distal regions of *Ae. tauschii* chromosomes [17] is largely due to shortening of inter-insular distances.

Insulae in the distal gene-rich regions of wheat chromosome 3B were reported to contain larger numbers of genes than insulae in the proximal, gene-poor chromosome regions [16]. This was not observed in the *Ae. tauschii* genome. The two-fold larger threshold assumed to delimit intra- and inter-insular spaces in this study (81 kb) compared to the 3B study (43 kb), and the exclusion of pseudogenes from our analysis could have caused this difference. Our observation that insulae contain similar numbers of genes in distal chromosome regions compared to the less gene-dense proximal chromosome regions is paralleled by similar numbers of genes per insula in the genome of sorghum (3.7 genes per insula) compared to the less gene-dense genome of *Ae. tauschii* (3.9 genes per insula).

The observation that the greater gene density in the distal regions of chromosome 3B is due to genes that are not syntenic with rice and *B. distachyon* [16] is probably unrelated to the insular dynamics. It is more likely a reflection of a high proportion of novel genes in those regions [17], [32], [33] due to greater incidence of duplicated genes in the distal regions of wheat chromosomes and those of wheat diploid ancestors [17], [32].

If the evolution of gene insulae was driven entirely by insertions of LTR retroelements, it would be counter-intuitive to expect conservation of insulae over long periods of time. This is particularly true if the remarkably high rate of turnover of intergenic spaces in grass genomes is taken into account. In the Triticeae genomes, for example, virtually the entire intergenic space equivalent to nearly 90% of the genome, is replaced within four to five million years [34]. The four species studied here belong to grass subfamilies Pooideae (*Ae. tauschii* and *B. distachyon*), Ehrhartoideae (rice) and Panicoideae (sorghum). The Panicoideae subfamily diverged about 52.5 million years ago (MYA) from the ancestor of the Pooideae and Ehrhartoideae subfamilies [9]. Within the subfamily Pooideae, *Ae. tauschii* and *B. distachyon* diverged 35.8 MYA and their common ancestor diverged from rice 47.3 MYA [9]. In spite of the antiquity of these divergence times, comparison of the four genomes suggested that heterogeneity in gene distribution is conserved to some degree. Neighboring genes that are close to each other in one genome tend to be close to each other in other genomes and, *vice versa*, neighboring genes that are far apart in one genome show a tendency to be far apart in other genomes. This observation remained true even when comparing intergenic distances in *Ae. tauschii* with those in maize (Table S5), a Panicoideae species with a haploid genome size of 2,500 Mb (*P*-value <0.005).

The evolutionary conservation of gene distribution over long spans of time argues for a functional factor playing a role in the evolution of insulae, in addition to the process based on the dynamics of LTR retroelement insertions. It is possible that some gene pairs do not tolerate separation by LTR retroelements. Co-location of functionally related or co-expressed genes or genes encoding proteins in the same biochemical pathways was reported in a number of plants [16], [35]–[39]. Such functional constrains may represent another factor playing a role in the formation and conservation of insulae.

## Supporting Information

Table S1 **Locations of genes in nine** ***Ae. tauschii*** **contigs, the distances between neighboring genes, and homology of intergenic regions to LTR retrotransposons.**
(XLSX)

Table S2 **Locations of genes in the rice regions orthologous to the** ***Ae. tauschii*** **contigs, the distances between neighboring rice genes, and homology of the intergenic regions to LTR retrotransposons.**
(XLSX)

Table S3 **Locations of genes in the** ***B. distachyon*** **regions orthologous to the** ***Ae. tauschii*** **contigs and the distances between neighboring** ***B. distachyon*** **genes.**
(XLSX)

Table S4 **Locations of genes in the** ***S. bicolor*** **regions orthologous to nine** ***Ae. tauschii*** **contigs and distances between neighboring** ***S. bicolor*** **genes.**
(XLSX)

Table S5 **Orthologous gene pairs and their intergenic distances in** ***Ae. tauschii*** **, rice,** ***B. distachyon*** **, sorghum, and maize.**
(XLSX)

Table S6 **Orthologous gene pairs in** ***Ae. tauschii*** **and rice, their intergenic distances and homology of the intergenic sequences to known retrotransposons.**
(XLSX)
